# Association of elevated transcript levels of interferon-inducible chemokines with disease activity and organ damage in systemic lupus erythematosus patients

**DOI:** 10.1186/ar2510

**Published:** 2008-09-15

**Authors:** Qiong Fu, Xiaoqing Chen, Huijuan Cui, Yanzhi Guo, Jing Chen, Nan Shen, Chunde Bao

**Affiliations:** 1 Shanghai Institute of Rheumatology, Renji Hospital, Shanghai Jiao Tong University School of Medicine, Shan Dong Middle Road, Shanghai 200001, PR China; 2 Molecular Rheumatology Laboratory, Institute of Health Sciences, Shanghai Institutes for Biological Sciences, Chinese Academy of Sciences and Shanghai Jiao Tong University School of Medicine, Chong Qing South Road, Shanghai 200025, PR China

## Abstract

**Introduction:**

Systemic lupus erythematosus (SLE) is a multi-system autoimmune disease with a heterogeneous course and varying degrees of severity and organ damage; thus, there is increasing interest in identifying biomarkers for SLE. In this study we correlated the combined expression level of multiple interferon-inducible chemokines with disease activity, degree of organ damage and clinical features in SLE, and we investigated their roles as biomarkers.

**Methods:**

Peripheral blood cells obtained from 67 patients with SLE patients, 20 patients with rheumatoid arthritis (RA) and 23 healthy donors were subjected to real-time PCR in order to measure the transcriptional levels of seven interferon-inducible chemokines (RANTES, MCP-1, CCL19, MIG, IP-10, CXCL11, and IL-8). The data were used to calculate a chemokine score for each participant, after which comparisons were performed between various groups of SLE patients and control individuals.

**Results:**

Chemokine scores were significantly elevated in SLE patients versus RA patients and healthy donors (*P *= 0.012 and *P *= 0.002, respectively). Chemokine scores were correlated positively with SLE Disease Activity Index 2000 scores (*P *= 0.005) and negatively with C3 levels (*P *< 0.001). Compared with patients without lupus nephritis and those with inactive lupus nephritis, chemokine scores were elevated in patients with active lupus nephritis, especially when their daily prednisone dosage was under 30 mg (*P *= 0.002 and *P *= 0.014, respectively). Elevated chemokine scores were also associated with the presence of cumulative organ damage (Systemic Lupus International Collaborating Clinics/American Society of Rheumatology Damage Index ≥ 1; *P *= 0.010) and the occurrence of anti-Sm or anti-RNP autoantibodies (both *P *= 0.021).

**Conclusions:**

The combined transcription level of interferon-inducible chemokines in peripheral blood leucocytes is closely associated with disease activity, degree of organ damage, and specific autoantibody patterns in SLE. The chemokine score may serve as a new biomarker for active and severe disease in SLE.

## Introduction

Systemic lupus erythematosus (SLE) is a multi-system autoimmune disease characterized by immune dysregulation that results in the production of antinuclear and other autoantibodies, as well as immune complex deposition in the kidneys and other organs. The disease course of SLE is heterogeneous and characterized by unpredictable flares and remissions. Thus, there is a pressing need to identify biomarkers that will facilitate better assessment of disease activity and organ involvement, and provide insight into the relationships between pathogenesis and clinical manifestations.

Recently, we and others have used gene expression microarrays to identify a group of type I IFN-inducible genes (IFIGs) that are significantly upregulated in peripheral blood cells from SLE patients [1-4]. The expression of these IFIGs, often referred to as IFN signatures, was later found to be closely associated with increased disease activity, specific autoantibody profiles and significant organ damage in SLE patients [5,6]. In addition to carrying markers of the IFN signature, peripheral blood cells from SLE patients are also elevated in a variety of chemokines [7]. Chemokines are a group of small molecules with the ability to recruit specific leucocytes to target tissue sites, thereby contributing to the organ damage seen in SLE. Other functions of chemokines include their ability to influence dendritic cell maturation, induction of B-cell and T-cell development, determination of peripheral cell localization, and involvement in T-helper-1 and T-helper-2 polarization [8].

A number of studies have identified increased plasma concentrations of chemokines, including 'regulated upon activation normal T-cell expressed and secreted' (RANTES), monocyte chemotactic protein (MCP)-1, IL-8, IFN-inducible protein 10 (IP-10), and monokine induced by IFN-γ (MIG), in patients with active SLE [9-12]. In addition, the *ex vivo *production of chemokines by peripheral blood cells from SLE patients appears to be significantly higher than that of cells from normal control individuals, after stimulation by lipopolysaccharide or phytohaemagglutinin [10], which suggests that the elevated expression of chemokines is involved in the immune dysregulation seen in this disorder.

Although the contributions made by chemokines in the pathogenesis of SLE have been studied extensively, the mechanisms that give rise to the increased chemokine responses in peripheral blood cells from SLE patients remain unclear. It has been reported that certain chemokine responses are strongly dependent upon IL-2 [13]. Another study [10] revealed that the plasma concentrations of IP-10 and MIG are significantly correlated with that of IL-18. A recent study [9] found that several serum chemokines were significantly elevated in SLE patients with increased expression of IFIGs, implying that the production of certain chemokines may be regulated by the type I IFN pathway. It is also interesting that IFN-inducible chemokines are significantly elevated in active SLE patients, a fact that raises the possibility that they might serve as novel biomarkers for SLE disease activity, and which adds a new link between these two essential aspects of SLE pathogenesis. However, the associations between the IFN-inducible chemokines and the clinical features of SLE have not been fully studied. Moreover, the finding that IFN-inducible chemokines may serve as a biomarker in active SLE requires verification in a larger cohort of patients, as well as in patients from different races and backgrounds.

In the present study we measured the transcription levels of seven IFN-inducible chemokines, as well as those of five classical IFIGs, in peripheral blood cells drawn from 67 patients with SLE, 20 with rheumatoid arthritis (RA), and 23 healthy donors, and calculated a chemokine score and an IFN score for each participant. We found that the transcriptional levels of IFN-inducible chemokines in peripheral blood cells were closely associated with disease activity and organ damage in SLE, and may be useful in disease monitoring and prognostication.

## Materials and methods

### Patients and control individuals

This study was approved by the Review Board for RenJi Hospital inShanghai, Republic of China. Informed consent was obtained from all study participants. All studies were performed in accordance with the Declaration of Helsinki. Sixty-seven Chinese patients with SLE, 20 with RA, and 23 age-matched and sex-matched healthy donors were enrolled in the study (Table 1). The SLE and RA patients fulfilled the classification criteria of the American College of Rheumatology for SLE [14] and RA [15], respectively. All SLE and RA patients were recruited from the Lupus Clinic Center of RenJi Hospital, Shanghai JiaoTong University School of Medicine. Healthy donors were selected from a pool of healthy volunteers at the RenJi Hospital, aiming to match them to the lupus patients with respect to age and sex. Otherwise eligible individuals with a current or recent infection were excluded from the study.

**Table 1:** Demographics of SLE and RA patients and healthy donors

	SLE patients (n = 67)	RA patients (n = 20)	Healthy donors (n = 23)
Age (years)	35.43 ± 1.85 (14–60)	37.2 ± 1.68 (17–61)	32.21 ± 2 (16–58)
Sex (%)			
Female	89.6	85	83.3
Male	10.4	15	16.7
Disease duration (years)	5.58 ± 0.75 (0.04–24)	5.93 ± 1.3 (1.2–21)	-
ANA (%)	95.5	37.1	-
SLEDAI-2K	8.06 ± 0.68 (0–25)	-	-
SDI	0.82 ± 0.17 (0–6)	-	-

Except where otherwise indicated, values are expressed as mean ± standard error of the mean (range). There were no significant differences between patients with SLE, patients with RA and healthy donors in terms of age and sex. ANA, antinuclear antibody; RA, rheumatoid arthritis; SDI, Systemic Lupus International Collaborating Clinics/American College of Rheumatology Damage Index; SLE, systemic lupus erythematosus; SLEDAI-2K, SLE Disease Activity Index 2000.

The lupus patients were all receiving steroid therapy at the time of the study, with an average prednisone (or equivalent) dosage of 40 mg/day. In addition, 28 patients were taking immunosuppressive therapy and 28 were receiving an antimalarial drug (hydrochloroquine 200 to 400 mg/day). For each patient, disease activity and disease-related damage were assessed at the time of blood donation using the SLE Disease Activity Index 2000 (SLEDAI-2K) [16] and the Systemic Lupus International Collaborating Clinics/American College of Rheumatology Damage Index (SDI) [17].

### Sample handling and RNA processing

Peripheral blood samples donated by each participant were collected in tubes containing anticoagulant-citrate-dextrose solution A. After plasma was collected, erythrocytes were lysed immediately and total RNA extracted from leucocytes using Trizol Reagent (Invitrogen, Carlsbad, CA, USA). Traces of DNA contamination were routinely removed by On-column DNase treatment using RNeasy Mini Kit (Qiagen, Hamburg, Germany). The integrity of RNA was assessed using capillary gel electrophoresis, and the quality and quantity of RNA were measured using NanoDrop™ 1000 Spectrophotometer (NanoDrop Technologies, Wilmington, DE, USA) with 260 nm/280 nm ratio above 1.8. About 1 μg total RNA was then reverse transcribed into cDNA using SuperScript II Reverse Transcriptase (Invitrogen). All plasma, RNA and cDNA samples were stored at -70°C before use.

### Real-time PCR

To quantify the expression of genes encoding IFN-inducible chemokines and IFIGs, the transcriptional levels of a total of seven IFN-inducible chemokine genes (RANTES, MCP-1, MIG, IP-10, C-X-C chemokine ligand [CXCL]11, IL-8, and C-C chemokine ligand [CCL]19) and five IFIGs (IFN-induced protein with tetratricopeptide repeats [IFIT]1, IFIT3, myxovirus resistance 1 [Mx1], oligoadenylate synthetase [OAS]1, and lymphocyte antigen 6 complex, locus E [Ly6e]) were measured by real-time PCR using SYBR Green. All cDNA samples were amplified in duplicate using Premix Ex Taq™ (Takara, Shiga, Japan), with the expression of ribosomal protein L13a used as an internal control for each sample. Details of the method were described previously [2,18]. Primer sequences are given in Table 2.

**Table 2:** Primers used to amplify transcripts of chemokines and IFIGs

Gene	Forward	Reverse
RPL13A	5'-CTGGAGGAGAAGAGGAAAGA-3'	5'-TTGAGGACCTCTGTGTATTTGTCA-3'
Ly6e	5'-CTTACGGTCCAACATCAGAC-3'	5'-GCACACATCCCTACTGACAC-3'
OAS-1	5'-GAAGGCAGCTCACGA AAC-3'	5'-TTCTTAAAGCATGGGTAATTC-3'
Mx1	5'-GGGTAGCCA CTGGACTGA-3'	5'-AGGTGGAGCGATTCTGAG-3'
IFIT1	5'-TCAAAGTCAGCAGCCAGTCTCA-3'	5'-GCCTCCTTGGGTTCGTCTATAA-3'
IFIT3	5'-AACTACGCCTGGGTCTACTATCACTT-3'	5'-GCCCTTTCATTTCTTCCACAC-3'
RANTES	5'-CGCTGTCATCCTCATTGCTAC-3'	5'-GGGTGACAAAGACGACTGCT-3'
MCP-1	5'-CATTGTGGCCAAGGAGATCTG-3'	5'-CTTCGGAGTTTGGGTTTGCTT-3'
MIG	5'-GAGTGCAAGGAACCCCAGTAGT-3'	5'-TTGTAGGTGGATAGTCCCTTGGTT-3'
IP-10	5'-TTCAAGGAGTACCTCTCTCTAG-3'	5'-CTGGATTCAGACATCTCTTCTC-3'
CXCL11	5'-CAAACATGAGTGTGAAGGGC-3'	5'-ATGCAAAGACAGCGTCCTCT-3'
CCL19	5'-CCTGCTGGTTCTCTGGACTT-3'	5'-CTCACGATGTACCCAGGGAT-3'
IL-8	5'-TGCCAAGGAGTGCTAAAG-3'	5'-CTCCACAACCCTCTGCAC-3'

CCL, C-C chemokine ligand; CXCL, C-X-C chemokine ligand; IFIG, IFN-inducible gene; IFIT, interferon-induced protein with tetratricopeptide repeats; IL, interleukin; IP-10, interferon-inducible protein 10; Ly6e, lymphocyte antigen 6 complex, locus E; MCP, monocyte chemotactic protein; MIG, monokine induced by interferon-γ; Mx1, myxovirus resistance 1; OAS, oligoadenylate synthetase; RANTES, regulated upon activation normal T-cell expressed and secreted.

### Calculation of chemokine scores and IFN scores

IFN scores were calculated as described in previous studies [5,6]. The mean and standard deviation (SD) for the expression level of each IFIG in the healthy donor group (mean_HD _and SD_HD_, respectively) were used to obtain a standardized expression level (S) of each gene for each SLE patient, as follows: S = (Gene_SLE _- Gene_HD_)/SD (Gene_HD_). In this equation, Gene_SLE _is the expression level of a particular gene in a given SLE patient and Gene_HD _is the mean level of this gene in healthy donors. All of the standardized expression level values were summed to calculate a total IFN expression score for each participant [5]. A chemokine score for each participant was calculated in a similar manner.

### Statistical analysis

Data were analyzed using the SPSS software for Windows (Version 11.0; SPSS Inc., Chicago, IL, USA). The continuous variable data were not normally distributed because of the extremely elevated expression of IFIGs and chemokines in particular patients; consequently, all values were expressed as medians with 25th and 75th percentiles and interquartile ranges (IQRs), and comparisons were conducted using the nonparametric Mann-Whitney test. Correlations between groups were evaluated using the Spearman test. *P *values under 0.05 were considered statistically significant.

## Results

### Increased average chemokine score in SLE patients

**Figure 1:**
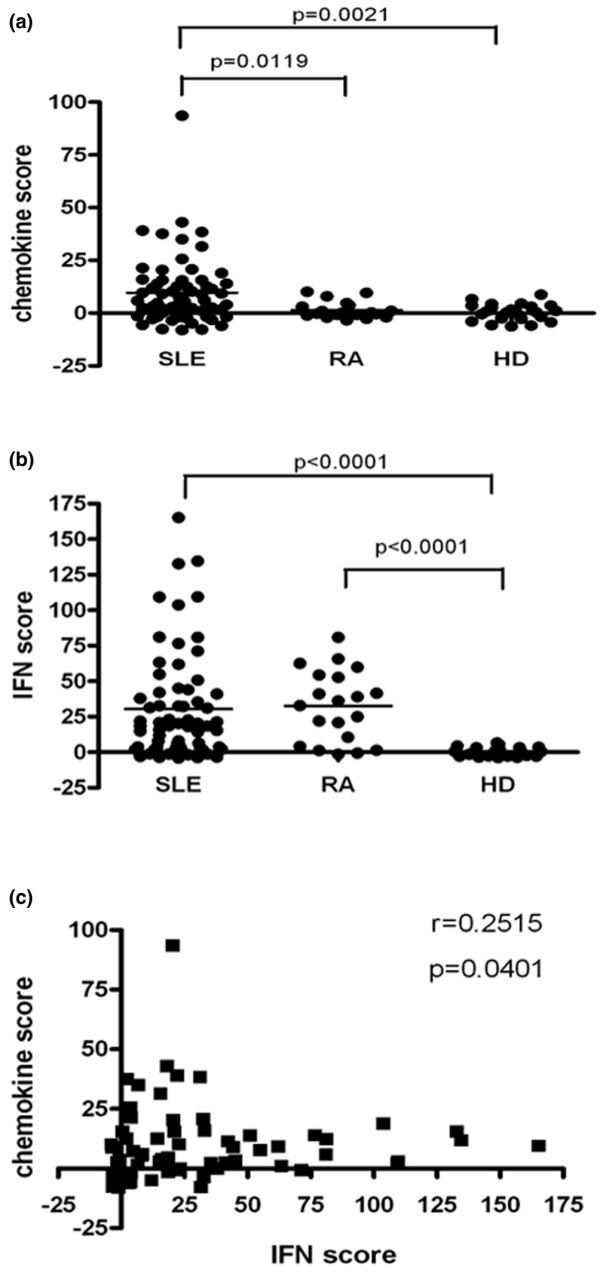
Comparison of chemokine and IFN scores between SLE and RA patients, and healthy donors. The methods employed to calculate the chemokine score and the IFN score are described in Materials and methods. **(a) **Chemokine scores were significantly elevated in SLE patients versus RA patients and healthy donors. **(b) **IFN scores were significantly elevated both in SLE and RA patients versus healthy donors. **(c) **Chemokine scores were positively correlated with IFN scores in SLE patients. Each symbol represents an individual patient; horizontal lines indicate median values. IFN, interferon; RA, rheumatoid arthritis; SLE, systemic lupus erythematosus.

The expression of seven IFN-inducible chemokine genes (RANTES, MCP-1, MIG, IP-10, CXCL11, IL-8 and CCL19) and five classic IFIGs (IFIT1, IFIT3, Mx1, OAS1 and Ly6e) in peripheral blood cells from 67 SLE patients, 20 RA patients and 23 healthy donors were measured using real-time reverse transcription PCR. SLE patients, RA patients, and healthy donors did not differ significantly with respect to mean age or sex distribution (Table 1). In general, the lupus patients had moderate disease activity and severity, with a mean SLEDAI-2K score of 8.06 and mean SDI of 0.82 (Table 1). As shown in Figure 1a, SLE patients had significantly higher chemokine scores than did either RA patients or healthy donors (*P *= 0.012 and P = 0.002, respectively). There was no increase in chemokine score in RA patients relative to healthy donors; however, when classic IFN scores were considered, there were no significant differences between the SLE and RA patients, although the scores from both groups were notably elevated compared with those of healthy donors (*P *< 0.001; Figure 1b). In addition, chemokine scores were significantly correlated with IFN scores in SLE patients (P = 0.040; Figure 1c). These data demonstrated relatively coordinated chemokine and IFN scores in SLE patients but a discrepancy between the scores in RA patients.

### Correlation of chemokine score with disease activity, as assessed using SLEDAI-2K and hypocomplementaemia

**Figure 2:**
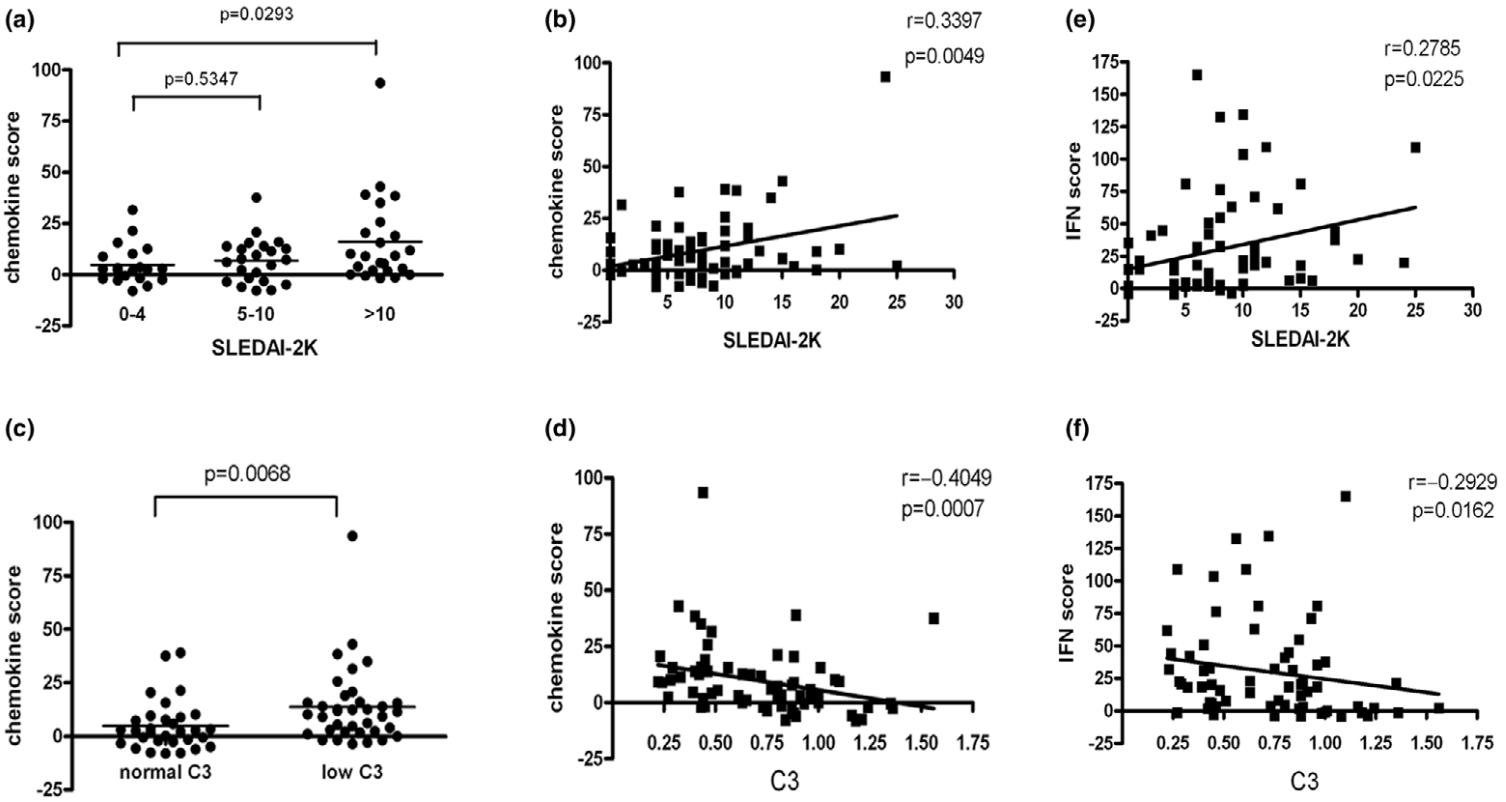
Association of chemokine and IFN scores with disease activity in SLE patients. Each symbol represents an individual patient; horizontal lines indicate median values. **(a) **SLE patients with a moderate-to-severe flare of disease (SLEDAI-2K score > 10) had significantly higher chemokine scores than did those without a disease flare (SLEDAI-2K score < 4) at the time of blood donation. **(b) **Chemokine scores were positively correlated with SLEDAI-2K. **(c) **Chemokine scores were significantly elevated in SLE patients with a reduced level of complement C3 (<80 mg/dl) compared with those with normal levels of C3. **(d) **A significantly negative correlation was observed between the chemokine score and C3 level. In addition, IFN scores were also correlated **(e) **positively with SLEDAI-2K and **(f) **negatively with C3 level. IFN, interferon; RA, rheumatoid arthritis; SLE, systemic lupus erythematosus; SLEDAI-2K, SLE Disease Activity Index 2000.

To investigate whether the expression of IFN-inducible chemokines might be related to SLE disease activity, we compared chemokine scores in SLE patients with different levels of disease activity, as assessed using SLEDAI-2K and the level of complement C3. SLE patients were divided into those with stable disease (SLEDAI-2K score 0 to 4), those with a mild flare (SLEDAI-2K score 5 to 10) and those with a moderate to severe disease flare (SLEDAI-2K score > 10), in accordance with the SLEDAI-2K flare system. We found that chemokine scores were significantly greater in SLE patients with a moderate to severe flare of disease than in patients without a flare (*P *= 0.029; Figure 2a). Chemokine scores were positively correlated with SLEDAI-2K scores (*r *= 0.34, *P *< 0.005; Figure 2b). C3 level is also an indicator of disease activity, with a low C3 level often observed in SLE patients with active disease. SLE patients with low C3 levels in the present study had a notably higher mean chemokine score than did those with normal C3 (*P *= 0.007; Figure 2c). Further analysis identified a negative correlation between chemokine scores and C3 level (*r *= 0.41, *P *< 0.001) (Figure 2d). The IFN score also exhibited notable correlations with SLEDAI-2K score and C3 level. However, it appeared not to attain the same level of significance as the chemokine score (*P *= 0.023 versus *P *< 0.005 and *P *= 0.016 versus *P *< 0.001, respectively; Figure 2e,f).

When other indicators of disease activity were considered, the chemokine and IFN scores were not significantly correlated with ESR or circulating levels of IgG anti-dsDNA antibody (data not shown). There were no differences in the mean value of chemokine scores between SLE patients with or without rash or arthritis (Table 3).

**Table 3:** Chemokine scores by presence or absence of SLE clinical features

Clinical features	SLE clinical features present		SLE clinical features absent		*P*
	n	Median (interquartile range)	n	Median (interquartile range)	
Renal	36	8.32 (1.43 to 19.41)	31	3.39 (-1.43 to +12.48)	NS
Neurological	4	1.27 (-0.65 to +4.17)	63	6.23 (-0.42 to +15.60)	NS
Arthritis	12	5.09 (-2.48 to +13.98)	55	5.93 (0.12 to 14.01)	NS
Serositis	13	5.47 (0.67 to 38.08)	54	5.32 (-0.93 to +13.96)	NS
Rash	22	2.37 (-2.27 to +13.96)	45	7.39 (0.89 to 15.634)	NS
Mucosal ulcer	8	4.21 (1.95 to 14.33)	59	5.93 (-4.46 to +14.01)	NS
Haematological	19	7.39 (-2.77 to +13.91)	48	5.09 (0.59 to 14.81)	NS
Proteinuria	26	8.511 (2.01 to 23.25)	41	3.39 (-1.56 to +12.56)	NS
Autoantibodies					
Anti-dsDNA	37	5.47 (-0.32 to +14.17)	30	5.72 (-0.44 to +14.81)	NS
Anti-Ro	24	6.05 (1.85 to 13.96)	43	5.47 (-1.76 to +15.69)	NS
Anti-Sm	13	11.56 (3.89 to 23.82)	54	3.56 (-1.72 to +12.66)	0.0211
Anti-RNP	23	10.28 (3.08 to 18.97)	44	2.95 (-1.72 to +12.56)	0.0212
Anti-nucleosome	24	7.50 (1.63 to 15.69)	43	3.73 (-0.46 to +13.91)	NS
Medical therapy					
Predisone dose >30 mg/day	38	4.76 (-1.56 to +12.66)	29	7.72 (2.12 to 19.89)	NS
Immunosuppressants	28	9.98 (0.53 to 17.33)	39	3.08 (-1.43 to +12.65)	NS
CQ/HCQ	28	2.15 (-1.72 to +12.11)	39	8.92 (2.34 to 18.97)	0.0481

anti-dsDNA, anti-double-stranded DNA; CQ, chloroquine; HCQ, hydrochloroquine; NS, not significant; SLE, systemic lupus erythematosus.

### Elevated chemokine scores in SLE patients with organ damage

**Figure 3:**
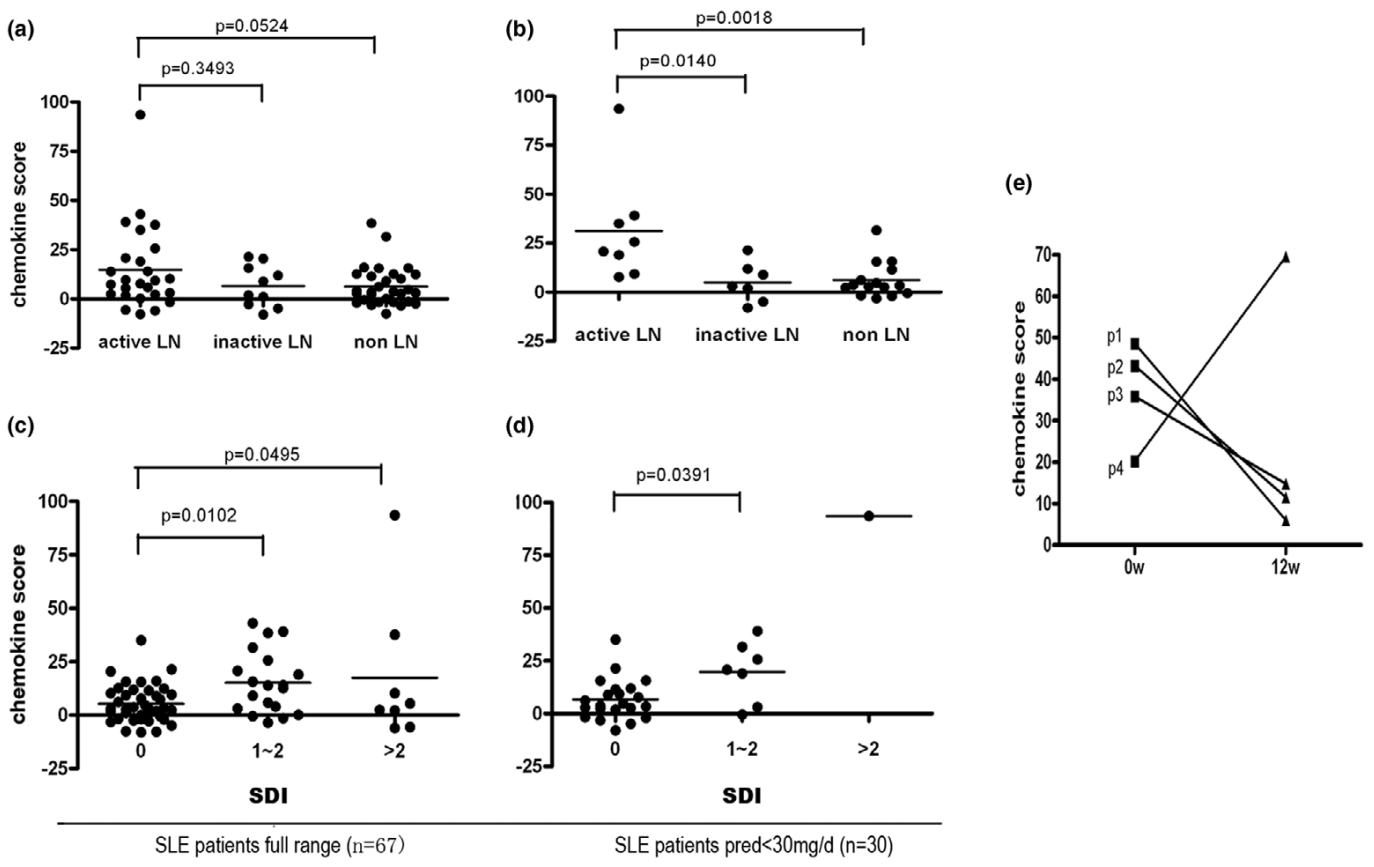
Elevated chemokine scores in SLE patients with organ damage. Each symbol represents an individual patient; horizontal lines indicate median values. **(a) **Chemokine scores exhibited a positive trend toward elevation in patients with active lupus nephritis (LN; n = 26) relative to patients with inactive LN (n = 10) and those with no history of LN (n = 31). **(b) **In the cohort, 30 patients were receiving daily doses of prednisone under 30 mg at the time of blood draw. Among them, eight patients had current LN, seven had inactive LN and 15 had never experienced renal manifestations of SLE. Patients with active renal disease had significantly higher chemokine scores than those with inactive LN or without LN. **(c) **Chemokine scores were significantly elevated in SLE patients with chronic and irreversible organ damage (SDI score 1 to 2 or more) compared with those with no damage. **(d) **Among those patients whose daily dosage of prednisone was less than 30 mg, chemokine scores were also significantly higher in those with versus those without chronic organ damage. **(e) **Chemokine scores were calculated in four active LN patients at the beginning of and after 12 weeks of treatment. In patient (p) 1, p2 and p3 (who achieved significant clinical improvement after treatment) chemokine scores were notably decreased, whereas in p4 (who had rapidly progressed into renal failure) chemokine score was dramatically increased. LN, lupus nephritis; SDI, Systemic Lupus International Collaborating Clinics/American Society of Rheumatology Damage Index; SLE, systemic lupus erythematosus.

Given that chemokines are involved in tissue damage and inflammation, we next explored whether chemokine scores are related to organ damage in SLE patients. Lupus nephritis (LN) is one of the most serious manifestations of SLE. In our cohort, nearly 55% of patients had either previous or current LN. Patients were considered to have active renal disease if proteinuria was above 0.5 mg/day, haematuria was above 5 red blood cells per high-power field, pyuria was above 5 white blood cells/high-power field, or cellular casts were present. Infection, kidney stones, or other causes of these urine findings were excluded. Chemokine scores among SLE patients with active LN exhibited a positive trend toward elevation versus those without LN (*P *= 0.05), but the same trend was not evident (*P *= 0.34) in a comparison against those with inactive LN (Figure 3a).

Because prednisone may impair the expression of IFIGs by peripheral blood cells[5], medication used by patients at the time of blood donation could interfere with current results. Consequently, in order to limit the potential influence of high-dose prednisone on chemokine expression, we then selected SLE patients taking daily prednisone doses less than 30 mg to examine further the association between chemokine scores and renal manifestations. As shown in Figure 3b, in these subgroups of patients chemokine scores were significantly higher in patients with active LN than in those with inactive LN or without LN (*P *= 0.014 and *P *= 0.002, respectively; Figure 3b), indicating that chemokine scores are associated with ongoing renal inflammation.

We also investigated the association between chemokine scores and both chronic and irreversible tissue damage in SLE, comparing scores between SLE patients with different levels of chronic damage, as assessed using SDI. Results revealed significantly elevated chemokine scores in SLE patients with SDI scores of 1 to 2 and those with scores above 2 versus those without tissue damage (*P *= 0.010 and *P *< 0.05, respectively; Figure 3c). When patients with a prednisone dose under 30 mg/day were selected, those with an SDI score of 1 to 2 had significantly higher chemokine scores than did those with no damage (*P *= 0.039). Although there was only one patient in this lower dose prednisone analysis with SDI above 2 (which therefore prevented statistical analysis), the chemokine score of this single patient (SDI score = 6) did appear inordinately high relative to all others (Figure 3d). These data suggest that chemokine scores are associated with cumulative organ damage in SLE, and that such a score might be useful in predicting long-term outcomes in SLE patients.

In order to investigate whether the chemokine score is responsive to treatment and changes over time in conjunction with disease activity, we selected four SLE patients who had initial onset of biopsy-proved type IV LN and collected peripheral blood samples at the beginning of treatment and after 3 months of treatment. Three of the patients (patient 1, 3 and 4) used high-dose predisone(1 mg/kg per day) plus monthly pulse of cyclophosphamide (0.8 g/month), whereas the other (patient 2) used predisone plus mycophenolate mofetil (1.5 g/day). After 12 weeks of treatment, two patients (patients 1 and 2) achieved clinical renal remission, with the urinary protein level dropping to less than 0.5 g/24 hour and their daily dosage of predisone tapering to 35 mg. Patient 3 also had great improvement in LN, with dramatic decreases in her urinary protein level (from 6.5 g/24 hours to 0.8 g/24 hours). In concordance with the clinical improvement in nephritis, chemokine scores in these three patients also significantly lowered. In contrast, patient 4 did not respond to therapy and progressed rapidly to renal failure despite aggressive treatment, including repeated pulses of glucocorticoid (500 mg intravenous methylprednisolone) and cyclophosphamide therapies. Three months after the first blood draw, the patient was suffering from severe oedema and ascites, as well as aggravated renal and heart failure. In parallel, the chemokine score in her peripheral blood leucocytes was dramatically elevated compared with baseline (Figure 3e). The patient's condition worsened rapidly and she died a month later. This result, although preliminary, suggests that escalation in chemokine score may predict an unfavourable outcome.

Although chemokine scores and IFN scores appear to be linked, we did not find significant differences in the mean value of IFN scores between patients with various levels of SDI (*P *= 0.27; data not shown). This finding added additional credence to the use of chemokine scores as a novel biomarker for SLE.

### Association of chemokine scores with clinical features in SLE

To assess associations between chemokine scores and clinical manifestations, autoantibody profiles and medication use, the chemokine scores were compared between patients with versus those without certain clinical features. We identified no significant differences in chemokine scores between patients with versus those without rash, mucosal ulcer, arthritis, serositis, and either neurological or haematological manifestations of SLE (Table 3). However, chemokine scores did appear to be associated with autoantibody production, being elevated in patients with anti-Sm antibodies (median = 11.56, IQR = 3.89 to 23.82;*P *= 0.021) or anti-RNP antibodies (median = 10.28, IQR = 3.08 to 18.97; *P *= 0.021; Table 3). In contrast to these results, the presence of anti-dsDNA or anti-Ro antibodies was not significantly associated with chemokine score (Table 3).

When medical therapies were considered, chemokine scores were significantly decreased in patients on antimalarial drugs at the time of blood donation (median = 2.15, IQR = -1.72 to +12.11; *P *= 0.048; Table 3). Chemokine scores also exhibited a trend toward being lower in patients receiving medium to high doses of prednisone (>30 mg/daay; median = 4.76, IQR = -1.56 to +12.66; *P *> 0.05). Treatment with immunosuppressive agents was not associated with elevated or depressed chemokine scores (Table 3).

## Discussion

In the present study, we selected seven IFN-inducible chemokines (RANTES, MCP-1, CCL19, MIG, IP-10, CXCL11 and IL-8), and we investigated the associations between their combined expression level and specific clinical features of SLE. Of these seven chemokines, MCP-1, RANTES and CCL19 are members of the CC family, and preferably recruit monocytes, macrophages, T cells and dendritic cells. In contrast, MIG, IP-10, CXCL11 and IL-8 are from the CXCL family, the first three of which are chemoattractants of activated T cells, whereas IL-8 is chemotactic for neutrophils [8]. All of these chemokines have been reported to have consensus sequences for IFN-responsive elements, including ISRE (IFN-stimulated responsive element), GAS (IFN-γ activation site) and IRF (interferon regulatory factor), within their gene promoter regions [19-22]. Consequently, the expression levels of these chemokines can be regulated by the IFN pathway, making them IFN inducible. These chemokines have been studied extensively, and their contributions to SLE have been confirmed by several different investigative teams [23-26].

Rather than focusing on individual chemokines, as most previous investigators have done, we investigated the expression of multiple chemokines and assessed the impact that overall chemokine expression has on SLE disease features. We measured the transcription levels of these chemokines in peripheral leucocytes and calculated a chemokine score by combining their expression levels. Given that there are various sources of serum chemokines, other than those produced by peripheral blood leucocytes, measurement of the mRNA levels of these chemokines in peripheral blood cells is possibly a direct indicator of the dysregulation of chemokine expression that exists in peripheral immune cells in patients with SLE. Moreover, the method is simple, inexpensive and has high throughput, making it a suitable approach to gaining an overview of the expression of multiple chemokines.

In the SLE patients included in the study, IFN score was significantly correlated with chemokine scores (Figure 1c), implying that expression levels of the IFN-inducible chemokines are associated with those of classical IFIGs in SLE. This result, however, was difficult to interpret because we did find elevated chemokine scores in some SLE patients with a low IFN score (IFN-low) and low chemokine scores in patients with a high IFN score (IFN-hi). In addition, we found that the overall chemokine score was significantly higher in SLE patients than in RA patients or healthy donors (Figure 1a), whereas IFN score was elevated in both of the disease groups compared to healthy donors. This result verifies previous reports that IFIGs are notably elevated in a subgroup of RA patients [27] but fails to identify any increase in the expression of IFN-inducible chemokines in RA, indicating that an elevated chemokine score might be more specific for SLE than for RA.

One of the potential explanations for the discrepancy between the expression of IFIGs and IFN-inducible chemokines is the highly complicated regulation of chemokine expression that exists in various diseases. Stimuli other than type I IFNs, such as IL-18 or IL-2, as suggested by previous studies [10,13], may be playing a role in driving the expression of chemokines in SLE. Moreover, medication used by the patients at the time of blood donation may elicit different responses in the expression of chemokines or IFIGs. The use of multiple drugs (and probably different drugs) by patients in the two patient groups might also complicate data interpretation. Nevertheless, regardless of the precise mechanism, these data suggest that the chemokine score we present here, although closely linked to IFN score, is an independent index for research and has novel and specific clinical significance.

In the present study we found that chemokine scores were associated with disease activity, as assessed using the SLEDAI-2K score and C3 level, and with ongoing or cumulative organ damage, as assessed based on the presence of active LN or SDI score in SLE patients. An elevated chemokine score may thus be helpful to identify SLE patient with active and severe disease. The preliminary longitudinal data also show that these chemokine scores are responsive to treatment and may change in conjunction with disease activity and severity, suggesting that chemokine score might be used to monitor disease progression and guide therapy. One of the weaknesses of the SLEDAI-2K score is its insensitivity in detecting improvement or worsening in a manifestation, because this can only be recorded as absent or present. For example, although patient 3 (see Figure 3e) had a dramatic decrease in urinary protein level from 6.5 g/24 hours to 0.8 g/24 hours, the SLEDAI-2K score failed to capture the improvement because she was still scored as positive in the proteinuria category. Her chemokine score, however, exhibited a significant decrease in concordance with the clinical improvement. This result, although limited and preliminary, lent further support to the chemokine score as a new and valuable marker of SLE disease activity and severity. However, prospective longitudinal studies with a larger sample size and more visits are needed to assess the role of chemokine score as a reliable biomarker in SLE.

Our conclusion that increased overall production of IFN-inducible chemokines by peripheral blood cells is important in the pathogenesis of SLE is supported by a number of published papers. Chemokines may contribute to SLE by recruiting immune and inflammatory cells to target tissues and by altering the normal trafficking and localization of certain populations of immune cells in the body; hence, they may impair the normal function of such cells. In cutaneous lupus erythematosus, MIG and IP-10 have been found to be significantly upregulated in inflamed skin and to help in the recruitment of plasmacytoid dendritic cells (pDCs), the major producers of type I IFN, to the skin [28]. This result could explain, at least in part, the observation that the number of pDCs is reduced in the peripheral blood of SLE patients [29], and that pDCs are recruited into and enriched within inflamed target tissues [30,31]. Moreover, ectopic expression of CCL19 can retain dendritic cells in target tissue and prevent their normal homing and migration to lymph nodes [32]. Previous investigators have reported that systemic over-expression of MCP-1 in mice can impair the homing and migration of monocytes to a localized MCP-1 gradient [33], and that MCP-1 may inhibit the normal differentiation of monocytes, which is possibly one of the mechanisms that is involved in autoimmunity [34]. In confirmation of these reports, our current data demonstrate that the overall production of IFN-inducible chemokines, as measured using a chemokine score, may serve as a useful indicator of the ongoing state of immune dysregulation in SLE.

In addition, in a small-scale study we also observed that the expression levels of those IFN-inducible chemokines were notably elevated in CD14^+ ^monocytes compared with T and B lymphocytes from SLE patients, indicating that monocytes might be more important contributors to the chemokine score than lymphocytes (data not shown). Therefore, the number as well as the activation state of the circulating monocytes might be a valuable clinical marker in SLE. In accordance with this assumption, it was recently reported [35] that activated renal macrophages are markers of disease onset and remission in LN, adding the possibility that active circulating monocytes might also be useful in disease monitoring in SLE. However, additional studies are needed in this area.

The patients with anti-Sm or anti-RNP autoantibodies had higher chemokine scores than did SLE patients without these two autoantibodies. An association of chemokine score with anti-Sm and anti-RNP antibodies is, to our knowledge, reported here for the first time. The underlying pathophysiological mechanism for this remains unknown. One possible explanation, however, is that the expression of IFN-inducible chemokines is somewhat linked to the IFN signature. The association between the IFN signature and anti-RNP autoantibodies was reported in earlier studies [5,6,36]. Although the mechanisms are unclear, it has been proposed that activation of pDCs by single-stranded or double-stranded RNA, through Toll-like receptors, might lead to the induction of type I IFN production and enhanced presentation of RNA-associated materials to autoreactive T and B cells. This, in turn, has the potential to cause upregulation of IFIGs and the appearance of anti-RNA-associated protein autoantibodies. Given that patients who are positive for anti-Sm or anti-RNP antibodies exhibit increased IFN scores, it is not surprising that such patients also have higher expressions of IFN-inducible chemokines and exhibit higher chemokine scores.

## Conclusion

The present study provides new evidence that IFN-inducible chemokine gene transcript levels in peripheral blood leucocytes may act as a new and reliable marker for disease activity and organ damage in human SLE. The data also suggest that the type I IFN system may contribute to SLE by modulating the expression of chemokines, linking these two networks in the pathogenesis of SLE. Additional studies are required to elucidate the highly complex interactions between IFIGs and chemokines, especially within the context of specific autoimmune diseases. The findings of such studies will shed new light on the dysregulation of the immune system and the involvement of inflammation in the initiation and perpetuation of autoimmunity.

## Abbreviations

CCL: C-C chemokine ligand; CXCL: C-X-C chemokine ligand; IFIG: IFN-inducible gene; IFIT: interferon-induced protein with tetratricopeptide repeats; IFN: interferon; IL: interleukin; IP-10: IFN-inducible protein 10; IQR: interquartile range; Ly6e: lymphocyte antigen 6 complex, locus E; MCP: monocyte chemotactic protein; MIG: monokine induced by IFN-γ; Mx1: myxovirus resistance 1; OAS: oligoadenylate synthetase; PCR: polymerase chain reactions; pDC: plasmacytoid dendritic cell; RANTES: regulated upon activation normal T-cell expressed and secreted; SD: standard deviation; SDI: Systemic Lupus International Collaborating Clinics/American College of Rheumatology Damage Index; SLE: systemic lupus erythematosus; SLEDAI-2K: SLE Disease Activity Index 2000.

## Competing interests

The authors declare that they have no competing interests.

## Authors' contributions

QF, NS and CB designed the study. YG and JC collected clinical data and blood samples. HC participated in RNA extraction and cDNA preparation. QF and XC performed real-time PCR and conducted data analysis. QF, NS and XC wrote the manuscript. CB and NS supervised the study. All authors read and approved the final manuscript.
